# Tryptophan metabolism is differently regulated between large and small dogs

**DOI:** 10.1007/s11357-019-00114-x

**Published:** 2019-11-29

**Authors:** Jessica M. Hoffman, J. Veronika Kiklevich, Marika Austad, ViLinh Tran, Dean P. Jones, Angela Royal, Carolyn Henry, Steven N. Austad

**Affiliations:** 1 Department of Biology, University of Alabama at Birmingham, 1300 University Blvd. CH464, Birmingham, AL 35294 USA; 2 Division of Pulmonary Allergy and Critical Care, Department of Medicine, Emory University, Atlanta, GA USA; 3 Clinical Biomarkers Laboratory, Department of Medicine, Emory University, Atlanta, GA USA; 4 Veterinary Medical Diagnostic Laboratory, University of Missouri, 900 E Campus Dr, Columbia, MO 65211 USA

**Keywords:** Dog, Metabolomics, Body size, Tryptophan metabolism

## Abstract

Companion dogs have recently been promoted as an animal model for the study of aging due to their similar disease profile to humans, the sophistication of health assessment and disease diagnosis, and the shared environments with their owners. In addition, dogs show an interesting life history trait pattern where smaller individuals are up to two-fold longer lived than their larger counterparts. While some of the mechanisms underlying this size and longevity trade-off are strongly suspected (i.e., growth hormone/IGF-I), there are likely a number of undiscovered mechanisms as well. Accordingly, we have completed a large-scale global metabolomic profiling of dogs encompassing a range of sizes and ages from three cities across the USA. We found a surprisingly strong location signal in the metabolome, stronger in fact than any signal related to age, breed, or sex. However, after controlling for the effects of location, tryptophan metabolism emerged as significantly associated with weight of the dogs, with small dogs having significantly higher levels of tryptophan pathway metabolites. Overall, our results point toward novel, testable hypotheses about the underlying physiological mechanisms that influence size and longevity in the companion dog and suggest that dogs may be useful in sorting out the complexities of the tryptophan metabolic network.

**Electronic supplementary material:**

The online version of this article (10.1007/s11357-019-00114-x) contains supplementary material, which is available to authorized users.

## Introduction

Humans are living longer than ever before in the 300-thousand-year history of our species. Consequently, we often spend our later years battling frailty, disability, and/or multiple life-threatening illnesses. To begin to understand the underlying aging processes that so often degrade the quality of later life, model laboratory organisms such as *Caenorhabditis elegans*, fruit flies, and mice are most commonly employed with an ultimate goal of discovering interventions that can be translated to improve and extend human health. However, translation of findings from these common laboratory species to effective interventions in humans has been uncertain (Cheon and Orsulic 2011; von Scheidt et al. 2017). Among the reasons for this limited therapeutic translatability are (1) overreliance on one or a few genetic backgrounds, (2) model species accumulate very different late-life pathologies compared with humans, and (3) they are typically maintained in highly controlled, unchanging, specific pathogen-free, benign environments that bear little resemblance to the range of environments that humans inhabit. To address these issues, the companion dog has recently been promoted as a more representative model of human health and longevity (e.g., Hoffman et al. 2018a; Kaeberlein et al. 2016). Compared with laboratory mice, for instance, companion dogs are genetically heterogeneous, experience an extraordinarily similar range of late-life pathologies as humans, and of course share the same environments as their owners. In addition, because of the sophistication of veterinary science, their health status can be exquisitely monitored and pathologies identified with precision possibly only second to that of humans (e.g., Jin et al. 2016). In this regard, dogs have been found to have age-related degenerative valve disease (Urfer et al. 2017a) as well as reduced oral health with aging (An et al. 2018), both similar to humans. In a similar vein, dogs have recently been proposed as a model to study lifespan extending interventions, and early work with rapamycin supplementation showed no negative health effects with a potential improvement in heart function in a small cohort of dogs (Urfer et al. 2017b). While these studies all suggest the power of the dog as a model of translational human aging, little is known about the molecular changes that occur with, and contribute to, aging in the dog.

In addition to their strengths as a model of human aging and longevity, dogs show a potentially highly informative pattern relating body size and longevity. Across mammalian species, larger species tend to be longer lived (Healy et al. 2014); however, within a species, like the companion dog, smaller individuals are longer lived. This pattern is seen in other mammalian species that show a significant size variation including mice and rats (Miller et al. 2002; Rollo 2002), horses (Tapprest et al. 2017), and possibly humans (He et al. 2014; Ma et al. 2017), although data conflict on this last point with some studies observing that taller individuals live longer (e.g., Brandts and van den Brandt 2019). However, no other species is known to show the body size–longevity relationship to the extent of the companion dog, in which a 50-fold variation in body mass is negatively associated with a 2-fold range in lifespan (Fleming et al. 2011; O'Neill et al. 2013). Thus, weight can be used an approximation of longevity, as smaller dogs are expected to live longer than larger ones.

The physiological parameters that contribute to the size-longevity trade-off seen in mammals is multi-faceted. Growth hormone (GH) and IGF-I signaling are almost certainly involved. In mice, it is well established that reducing GH signaling, either by lowering hormone availability or by disrupting its receptor, increases longevity and preserves multiple dimensions of health (Bartke 2005). Consistent with these findings, in dogs, GH and circulating IGF-1 levels are higher in large compared with medium-sized dog breeds (Favier et al. 2001; Greer et al. 2011). In addition to the GH/IGF axis, recent research using cultured fibroblasts from small and large dogs suggests that differences in mitochondrial metabolism and oxidative stress might contribute to the longevity differences seen across different-sized dogs. Cells from large dogs have significantly higher rates of glycolysis and DNA damage (Jimenez et al. 2018), as well as higher mitochondrial respiration rates (Nicholatos et al. 2019). However, many of the underlying mechanisms that contribute to longevity in the dog are still unknown. By investigating size–longevity differences, we can begin to develop novel hypotheses about healthy aging that potentially can be translated to human (and dog) life-extending interventions.

One method to identify molecular networks underlying complex physiological processes is metabolomics, the analysis of thousands of individual metabolites in an organism to understand how changes in metabolism are associated with specific phenotypes of interest. Metabolomics has been used extensively in model organisms to understand aging and longevity (e.g., Fuchs et al. 2010; Hoffman et al. 2014; Houtkooper et al. 2011), as well as non-human primates (Hoffman et al. 2018b; Hoffman et al. 2016) and humans (Darst et al. 2019; Menni et al. 2013). Previous research has suggested that individual dog breeds show different metabolomic profiles (Lloyd et al. 2016; Nicholatos et al. 2019; Viant et al. 2007) and that metabolomic profiles change in response to diet and obesity (Forster et al. 2018; Soder et al. 2017), as well as with specific diseases (e.g., Gookin et al. 2018; Hasegawa et al. 2014; Minamoto et al. 2015). Dogs of varying sizes have been shown to have different levels of circulating amino acids (Middleton et al. 2017), as well as different metabolomic profiles (Nicholatos et al. 2019).

Here, we present the largest metabolomics study to date in the companion dog with the goal of developing novel hypotheses about mechanisms of canine aging and longevity. We specifically look at the dog metabolome sampled at three different locations across the USA to help assess the impact of environmental heterogeneity.

## Methods

### Sample collection

Whole blood from animals undergoing unrelated procedures was collected in EDTA tubes from companion dogs in three locations between 2016 and 2018: (1) Birmingham, AL; (2) San Antonio, TX; and (3) Columbia, MO. Birmingham samples were collected from stray animals brought into the Jefferson County animal control facility and generally healthy animals seen at local veterinary clinics under the direction of JVK. San Antonio samples were collected by JVK and MA from generally healthy animals belonging to private owners brought to a local spay/neuter clinic. Columbia samples were collected as part of diagnostic workups for Veterinary Health Center patients, with sample processing coordinated by AR and CH. Individuals from this location included acute and chronically ill animals in addition to apparently healthy animals. A majority of samples from Birmingham and Columbia were from dogs fasted at least 4–6 h before blood collection. The San Antonio samples were from animals fasted overnight before the day of collection. During blood draw, demographic information for each dog were recorded: age, sex, sterilization status, body weight, and body condition score (BCS), a measure of obesity in the dog. Breed of dog was assigned either by the tending veterinarian or by the owner of the dog (Table S1). Age of stray animals from the Jefferson County animal control facility was estimated by JVK from a combination of bone development, and dental and ocular characteristics. Collection of blood samples from person-owned dogs was approved under UAB IACUC 21121and MU ACUC 8240.

After sample collection, tubes were stored at 4 °C. Plasma was extracted by centrifugation and then frozen at – 80 °C until plasma metabolite extraction. Samples from the three collection sites were randomized to minimize batch effects during metabolomics analyses and shipped on dry ice to the Clinical Biomarkers Laboratory, Emory University, for analysis.

### Metabolomics

High-resolution metabolomics (HRM) profiling was completed using standardized methods (Liu et al. 2016; Soltow et al. 2013) as follows. The 133 samples were analyzed in three batches consisting of 44, 44, and 45 samples, each prepared daily along with pooled human plasma (Qstd3) for quality control. For analysis prior to the first and after the last batch, an additional aliquot of National Institute of Standards and Technology Standard Reference Material 1950 (NIST SRM1950) was processed and analyzed identically to the samples. Aliquots were removed from storage at − 80 °C, thawed on ice, and 50 μL was treated with 100 μL of ice-cold LC-MS-grade acetonitrile. Extracts were then equilibrated for 30 min on ice, centrifuged (16,100×*g* at 4 °C) for 10 min to remove precipitated proteins, and clear supernatant was transferred to 250-μL autosampler vials and maintained at 4 °C until analysis (< 22 h).

Sample extracts were analyzed using liquid chromatography and Fourier transform high-resolution mass spectrometry on a Dionex Ultimate 3000, Orbitrap Fusion™ Tribrid™ Mass Spectrometer system (Thermo Scientific) operated at 120,000 resolution. The chromatography system was operated in a dual-pump configuration that enabled parallel analyte separation and column flushing. For each sample, 10 μL aliquots were analyzed in triplicate using hydrophilic interaction liquid chromatography (HILIC) with electrospray ionization (ESI) source operated in positive mode and reverse-phase chromatography (RPC) with ESI operated in negative mode. Analyte separation for HILIC was accomplished by a 2.1 mm × 50 mm × 2.5 μm Waters XBridge BEH Amide XP HILIC and an eluent gradient (A = water, B = acetonitrile, C = 2% formic acid) consisting of an initial 1.5-min period of 22.5% A, 75% B, and 2.5% C followed by a linear increase to 77.5% A, 20% B, and 2.5% C at 4 min and a final hold of 1 min. RPC separation was by 2.1 mm × 50 mm × 3 μm end-capped C_18_ column (Higgins) using an eluent gradient (A = water, B = acetonitrile, C = 10 mM ammonium acetate) consisting of an initial 1-min period of 60% A, 35% B, and 5% C, followed by a linear increase to 0% A, 95% B, and 5% C at 1.5 min and held for the remaining 3.5 min. The mobile phase flow rate for HILICpos was held at 0.350 mL/min for the first 1.5 min, and increased to 0.400 mL/min for the remaining of the run. C18neg mobile phase flow rate was held at 0.400 ml/min for the first 2 min and then increased to 0.500 ml/min for the remaining 3.0 min. Data were collected for a mass-to-charge ratio (*m/z*) range 85–1275. Probe temperature, capillary temperature, sweep gas, and S-Lens RF levels were maintained at 200 °C, 300 °C, 1 arbitrary units (AU), and 45, respectively, for both ESI polarities. Additional source tune settings were optimized for sensitivity using a standard mixture; positive tune settings for sheath gas, auxiliary gas, sweep gas, and spray voltage setting were 45 AU, 25 AU, 1 AU, and 3.5 kV, respectively; negative settings were 45 AU, 5 AU, 1 AU, and − 3.0 kV. Maximum C-trap injection times of 100 ms and automatic gain control target of 1 × 10^6^ for both polarities. During untargeted data acquisition, no exclusion or inclusion masses were selected, and data were acquired in MS^1^ mode only. Data were stored as .raw files and converted to CDF format using Xcalibur file converter software (Thermo Fisher, San Diego, CA) for further data processing. Peak detection, noise filtering, *m/z* and retention time alignment, feature quantification, and data quality filtering were performed using apLCMS (Yu et al. 2009) with xMSanalyzer (Uppal et al. 2013). Data were extracted as *m/z* features where a feature is defined by *m/z*, retention time, and integrated ion intensity.

As a confirmation of metabolite values in our dataset, we compared individual metabolite values with known standards (Qstd3 as described above). This was done by taking the known Qstd3 value and multiplying it by metabolite intensity of each canine sample divided by the mean Qstd3 value for that metabolite. This gave us a “true” concentration of the metabolite in the canine samples compared with a human reference.

### Data analysis

Metabolomics data analysis was completed in the statistical language R unless otherwise stated (R Core Team 2018). Positive and negative ion mode data were analyzed separately. All data were first log-transformed and centered and scaled to using the “scale” function in R. Metabolites that were missing from more than 15% of all samples were removed from the analysis. Dogs who were recorded as being under 1 year of age were removed from the analysis as final body size had not yet been attained.

Our initial interest was in determining the association between individual metabolites, weight, and age, controlling for the effects of sex and location. We also investigated the effect of sterilization status on the metabolome and found little effect. Thus, sterilization status was dropped from our final model. Significance was set with a false discovery rate (FDR) of *α* < 0.05 (Benjamini and Hochberg 1995). Metabolites that were found to be associated with weight or age were run through the program *mummichog* to determine metabolic pathways that were significantly different for each factor individually (Li et al. 2013). We did not control or look at the impact of breed in our model as there were over 20 breeds represented in our final dataset. Therefore, we did not have the power to assess breed-specific differences.

In addition to our individual metabolite analyses, we examined the associations of the entire metabolome with sex, age, and body weight. Principal components analysis (PCA) was performed using the ade4 package in R (Dray and Dufour 2007), for only those metabolites that were present in all samples.

## Results

**Fig. 1:**
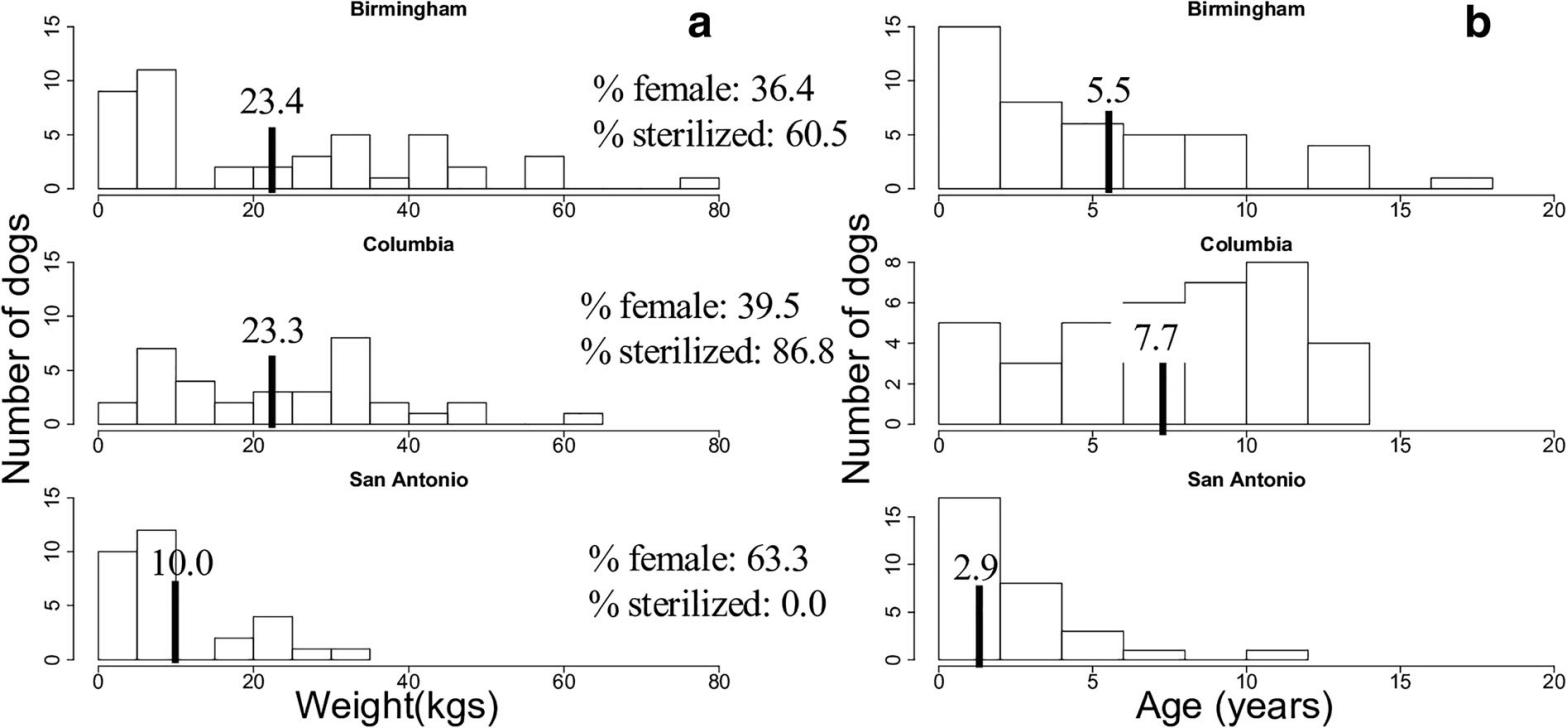
Profile of dogs sampled from the three locations. (**a**) Weight. (**b**) Age. Note that vertical lines indicate population mean. Percent of the sample that is female and that comes from sterilized dogs shown for each location

Our final dataset consisted of plasma samples from 112 dogs across the three locations (44-Birmingham, 38-Columbia, 30-San Antonio). The mean age of all animals from all sites was 5.5 years (1–17 years) with an average weight of 19.7 kg (2.1–76.4 kg). Males slightly outnumbered our female samples (62 males, 50 females); 49% of females and 55% of males were sterilized at the time of sample collection. Clear differences were noted in the characteristics of the dogs among the three collection sites (Fig. 1). Dogs from Columbia were overall older and largely sterilized compared with younger, smaller 100% intact dogs from San Antonio. In fact, dogs from San Antonio were smaller than those from either of the other two locations. In addition, 0%, 18.2%, and 71% of dogs at San Antonio, Birmingham, and Columbia had a serious health diagnosis at the time of sample draw (Table S1). Thus, the dogs sampled in Columbia were on average sicker than the other two populations. Therefore, all three locations represent very different canine populations.

Our final metabolomics dataset consisted of 6789 metabolites in the positive ion mode and 6614 metabolites in the negative ion mode. Our residual linear regression model and PCA comprised 3473 and 3442 metabolites in the positive and negative ion modes, respectively; this difference in metabolite number is due to including only those metabolites that were found to have no missing data across all samples in the analyses.

**Fig. 2:**
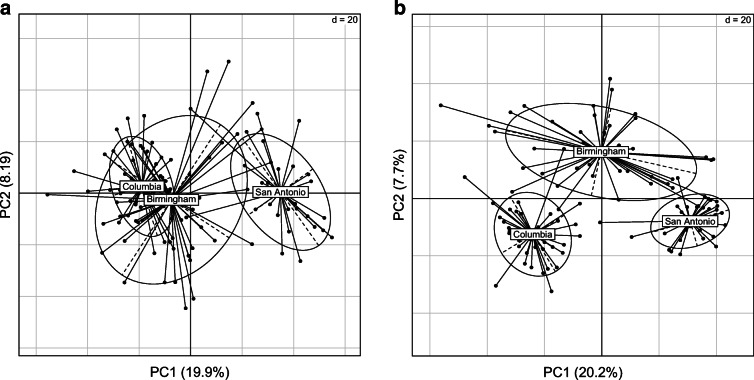
PCA effects of location for the **a** positive and **b** negative ion modes

We found that, perhaps not surprisingly, location had the biggest association with the metabolome. The metabolomes of San Antonio dogs were most different from Birmingham and Columbia (Figure 2). This location effect was so strong that it overwhelmed associations of individual metabolites with our factors of interest as little variation was left in the dataset (Table 1). To control this location effect, we utilized two different approaches. In the first, we took the residuals of each metabolite by location, and then used the residual values in a linear model. Secondly, we analyzed each location individually, comparing significant metabolites across locations.

**Table 1:** Number of metabolites associated with age, sex, and weight across all locations. Linear model has controlled for the effects of location. Models were run with 3473 and 3442 metabolites in the positive and negative ion modes, respectively (all metabolites with no missing data)

Column	Age	Sex	Weight
Hilic positive	161	1	59
C18 negative	293	11	32

**Fig. 3:**
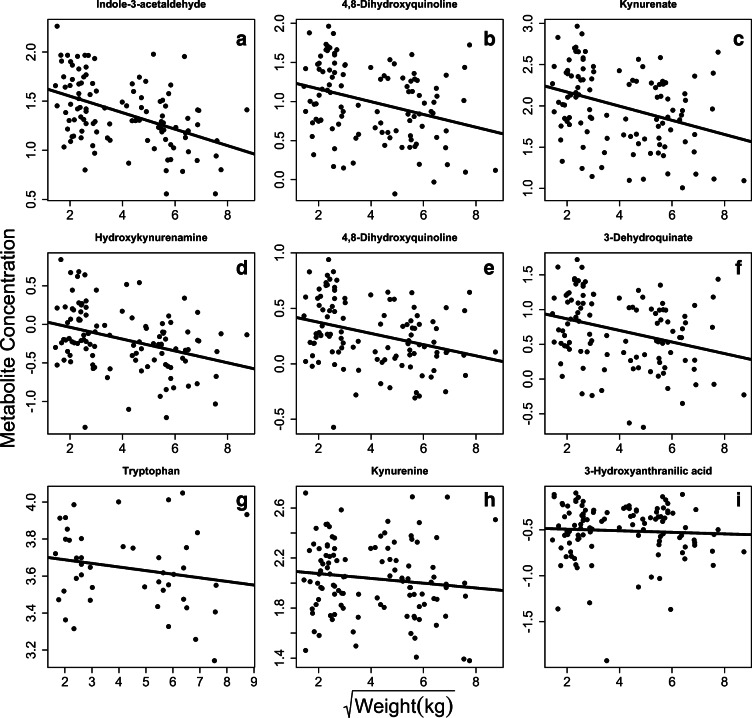
Tryptophan metabolites associated with weight across all location. **a–f** All were significantly associated with weight at FDR < 0.05. **g–i** Other annotated metabolites in the pathway that did not pass our FDR threshold. All significant metabolites are lower in larger dogs, controlling for the effects of age and sex. Note that weight has been square-root transformed to make visualization easier

Using the residuals of location, we were able to discover 161 and 293 metabolites associated with age and 59 and 32 metabolites associated with weight in the positive and negative ion modes, respectively (Table 2). We found very few metabolites associated with sex, 1 and 11 in the positive and negative modes, respectively. However, our PCA failed to separate the metabolome based on either age or weight (Figure S1). Metabolite enrichment analyses discovered 9 and 5 pathways associated with weight, and 8 and 31 pathways associated with age in the positive and negative ion modes, respectively (Table 3). We found a strong signal for differences in tryptophan metabolism between large and small dogs, with higher values of tryptophan metabolites always seen in the small dogs (Fig. 3a–f). However, tryptophan itself was not higher in small dogs (Fig. 3g), nor was kynurenine, its immediate metabolized breakdown product (Fig. 3h). We then quantified tryptophan using the raw intensity values and found that levels of tryptophan varied from 6.55–132.12 and showed a similar pattern with body weight and the normalized residual values in the model (Figure S2).

**Table 2:** Number of metabolites associated with age, sex, and weight for each location individually. Models were run with 6789 and 6614 metabolites in the positive and negative ion modes, respectively

Column	Location	Age	Sex	Weight
Hilic positive	Birmingham	930	0	106
	Columbia	0	0	0
	San Antonio	26	4	101
C18 negative	Birmingham	1474	0	90
	Columbia	0	0	0
	San Antonio	13	45	21

**Table 3:** Metabolic pathways associated with age and weight for Birmingham only (left) and all locations (right). Pathways in italics are associated with either age or weight in both analyses

Birmingham				All locations			
Column	Factor	Metabolic pathway	*p* value	Column	Factor	Metabolic pathway	*p* value
Positive	Weight	*Tryptophan metabolism*	0.0002	Positive	Weight	*Tryptophan metabolism*	0.0000
		Linoleate metabolism	0.0003			*Urea cycle/amino group metabolism*	0.0000
		*Urea cycle/amino group metabolism*	0.0003			*Xenobiotics metabolism*	0.0001
		*Arginine and Proline Metabolism*	0.0005			*Arginine and proline metabolism*	0.0001
		*Xenobiotics metabolism*	0.0008			Glycine, serine, alanine and threonine metabolism	0.0003
		Glycerophospholipid metabolism	0.0013			Drug metabolism - cytochrome P450	0.0006
		Vitamin B3 (nicotinate and nicotinamide) metabolism	0.0041			*Prostaglandin formation from arachidonate*	0.0007
		Tyrosine metabolism	0.0068			Aspartate and asparagine metabolism	0.0025
		De novo fatty acid biosynthesis	0.0073			Androgen and estrogen biosynthesis and metabolism	0.0325
		Glycosphingolipid metabolism	0.0092	Negative	Weight	Dynorphin metabolism	0.0008
		Pentose and Glucuronate Interconversions	0.0175			*Vitamin A (retinol) metabolism*	0.0064
		Alanine and Aspartate Metabolism	0.0364			*De novo fatty acid biosynthesis*	0.0075
		*Prostaglandin formation from arachidonate*	0.0427			Vitamin E metabolism	0.0193
		Purine metabolism	0.0427			Glycerophospholipid metabolism	0.0491
Negative	Weight	Phytanic acid peroxisomal oxidation	0.0043	Positive	Age	Vitamin B6 (pyridoxine) metabolism	0.0029
		*Vitamin A (retinol) metabolism*	0.0043			*Mono-unsaturated fatty acid beta-oxidation*	0.0066
		Urea cycle/amino group metabolism	0.0091			*Di-unsaturated fatty acid beta-oxidation*	0.0066
		Linoleate metabolism	0.0184			*Omega-6 fatty acid metabolism*	0.0143
		Fatty acid activation	0.0204			Tyrosine metabolism	0.0273
		*De novo fatty acid biosynthesis*	0.0271			Biopterin metabolism	0.0361
Positive	Age	Saturated fatty acids beta-oxidation	0.0015			Urea cycle/amino group metabolism	0.0400
		*Di-unsaturated fatty acid beta-oxidation*	0.0015			Alanine and aspartate metabolism	0.0486
		*Mono-unsaturated fatty acid beta-oxidation*	0.0019	Negative	Age	*Alanine and aspartate metabolism*	0.0002
		Purine metabolism	0.0021			*Arginine and proline metabolism*	0.0002
		Porphyrin metabolism	0.0022			*Urea cycle/amino group metabolism*	0.0002
		Fatty acid metabolism	0.0022			*TCA cycle*	0.0002
		Prostaglandin formation from dihomo gama-linoleic acid	0.0032			*Glutamate metabolism*	0.0002
		Alanine and aspartate metabolism	0.0033			*Beta-alanine metabolism*	0.0002
		Omega-6 fatty acid metabolism	0.0034			*Purine metabolism*	0.0002
		Dimethyl-branched-chain fatty acid mitochondrial beta-oxidation	0.0046			*Butanoate metabolism*	0.0002
		Phytanic acid peroxisomal oxidation	0.0058			*Pyrimidine metabolism*	0.0003
		De novo fatty acid biosynthesis	0.0105			Glycolysis and gluconeogenesis	0.0003
		Nitrogen metabolism	0.0121			Carbon fixation	0.0003
		*Omega-3 fatty acid metabolism*	0.0233			*Histidine metabolism*	0.0004
		*N*-Glycan degradation	0.0259			*Glycine, serine, alanine and threonine metabolism*	0.0004
		Heparan sulfate degradation	0.0259			*Aspartate and asparagine metabolism*	0.0005
		Chondroitin sulfate degradation	0.0259			Lysine metabolism	0.0006
		Lysine metabolism	0.0369			Valine, leucine and isoleucine degradation	0.0006
		Drug metabolism - other enzymes	0.0369			Fatty acid oxidation, peroxisome	0.0007
		Carbon fixation	0.0479			Tyrosine metabolism	0.0007
Negative	Age	*Arginine and proline metabolism*	0.0061			C5-Branched dibasic acid metabolism	0.0014
		*Pyrimidine metabolism*	0.0063			Methionine and cysteine metabolism	0.0022
		*Purine metabolism*	0.0064			**Phytanic acid peroxisomal oxidation**	0.0025
		*Alanine and aspartate metabolism*	0.0064			Nitrogen metabolism	0.0025
		*TCA cycle*	0.0066			Vitamin B1 (thiamin) metabolism	0.0025
		*Glycine, serine, alanine, and threonine metabolism*	0.0067			Propanoate metabolism	0.0049
		*Beta-Alanine metabolism*	0.0077			Pyruvate metabolism	0.0049
		*Histidine metabolism*	0.0082			Vitamin B6 (pyridoxine) metabolism	0.0066
		Ascorbate (vitamin C) and aldarate metabolism	0.0097			Glutathione metabolism	0.0101
		*Phytanic acid peroxisomal oxidation*	0.0108			*Glyoxylate and dicarboxylate metabolism*	0.0209
		*Urea cycle/amino group metabolism*	0.0147			Aminosugars metabolism	0.0287
		*Glycosphingolipid metabolism*	0.0168			*Glycerophospholipid metabolism*	0.0313
		*Butanoate metabolism*	0.0168			*Phosphatidylinositol phosphate metabolism*	0.0491
		*Phosphatidylinositol phosphate metabolism*	0.0225				
		*Glyoxylate and dicarboxylate metabolism*	0.0250				
		C5-Branched dibasic acid metabolism	0.0267				
		*Aspartate and asparagine metabolism*	0.0290				
		*Glutamate metabolism*	0.0316				
		Caffeine metabolism	0.0399				
		*N*-Glycan degradation	0.0413				
		Heparan sulfate degradation	0.0413				
		Chondroitin sulfate degradation	0.0413

When we looked within individual locations, we were actually able to find more metabolites associated with age and weight, even though our power was significantly decreased with the reduction in sample size in a location-specific analysis. Striking differences were seen in the numbers of metabolites associated with each factor of interest. Specifically, over 13% of the metabolome was associated with age in our Birmingham samples, but less than 1% were associated with age in San Antonio. This is not surprising as San Antonio had the smallest age variation (Fig. 1). No metabolites passed our FDR cutoff in our Columbia samples. Weight was associated with just over 1% of metabolites in both Birmingham and San Antonio, and again, no metabolites passed our FDR cutoff in Columbia. Sex was significantly associated with metabolite concentration only in the San Antonio location and only in the negative ion mode. The metabolites associated with age and weight in Birmingham and San Antonio rarely overlapped.

**Fig. 4:**
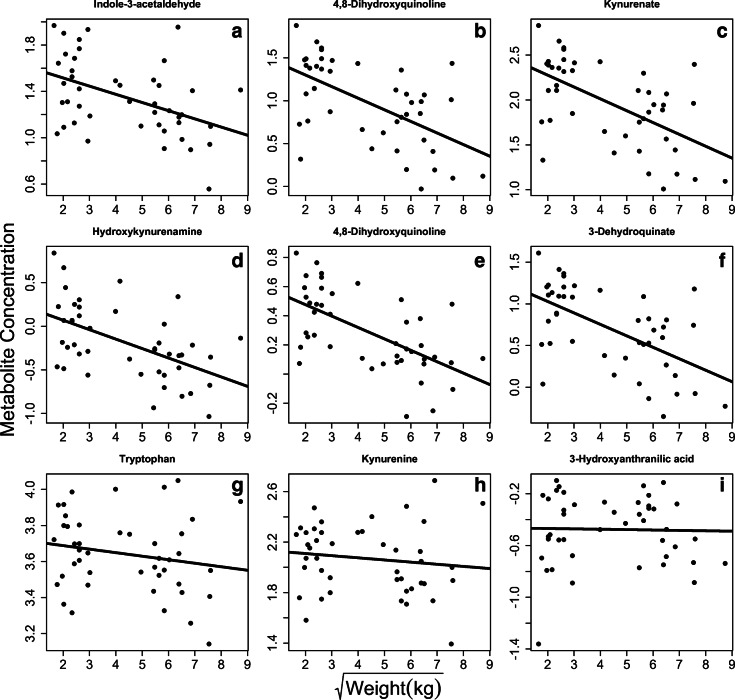
Tryptophan metabolites associated with weight in Birmingham only. **a–f** All were significantly associated with weight at FDR < 0.05. **g–i** Other annotated metabolites in the pathway that did not pass our FDR threshold. All significant metabolites are lower in larger dogs, controlling for the effects of age and sex. Note that weight has been square-root transformed to make visualization easier

For those metabolites that were associated with either weight or age in Birmingham, we ran metabolite enrichment analysis to determine which metabolic pathways were differentially associated with which variable. Similar to the entire combined datasets, the strongest differences between large and small dogs were related to tryptophan metabolism. Specifically, in the positive ion mode, 6 different metabolites were negatively associated with body size (Fig. 4). These findings replicate those seen in the location residual performed previously, suggesting many of the effects seen in the entire dataset are driven partially at least by the differences in the Birmingham location.

In addition to the differences in tryptophan metabolism, urea cycle metabolism, and metabolism of fatty acids, linoleate metabolism, de novo fatty acid synthesis, fatty acid activation, and vitamin A metabolism were associated with weight, while fatty acid metabolism and arginine, proline, and alanine metabolism were associated with age (Table 2). Many of the metabolic pathways associated with age and weight in the Birmingham sample were also significantly enriched in the entire dataset when using residuals of location as described above (Table 2).

**Fig. 5:**
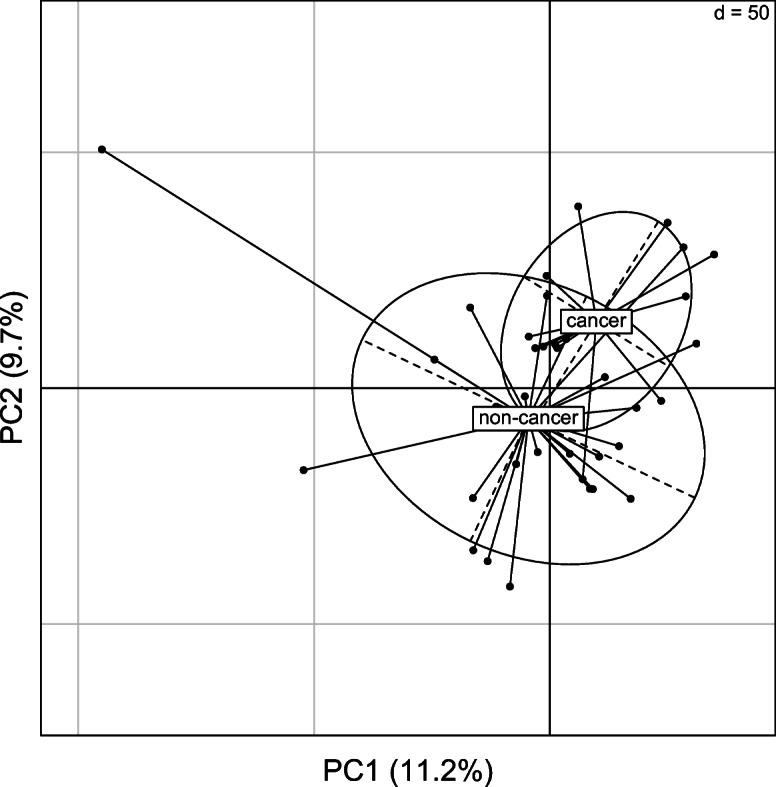
PCA of Columbia dogs with cancer status. Only the positive mode showed some separation of the two groups

Our Columbia population did not reveal any metabolites associated with age or weight. As the population was sicker than the Birmingham or San Antonio population, we were interested in understanding what might be driving the variation seen in this population. First, we divided the dogs into those that had a cancer diagnosis to those that either did not have cancer or had an unknown diagnosis. We found that at least in the positive ion mode, cancer status was able to somewhat separate the two group in a PCA analysis (Fig. 5). However, running a linear model with cancer status failed to find any metabolites that passed our FDR cutoff.

## Discussion

Here, we have completed the largest metabolomic profiling study to date in the companion dog, as well as the first to examine how the metabolome changes with age and weight. Interestingly, the largest effect we found was with regard to location of sampling. This large effect was quite unexpected, especially considering two of the locations (Birmingham and San Antonio) had samples drawn by the same veterinarian into EDTA tubes from the same shipment. However, the age, size, and reproductive status distributions of these two populations differed dramatically, so in retrospect the location effect was not as shocking. Columbia samples were collected in EDTA tubes not from the same shipment or brand as the Birmingham and San Antonio samples, yet animals in Columbia had a metabolome more similar to animals in Birmingham. This suggests that sample handling was most likely not the primary reason for the large location effect we witnessed. There are several potential explanations for these findings that immediately come to mind. First, as the dogs were residing in different locations, they were undoubtedly exposed to different gut microbiomes which could be influencing the circulating metabolome in the dogs. Secondly, there could be underlying viruses that do not affect the dog healthwise but do have some physiological effect on the metabolome. This specifically might explain the large differences observed in the San Antonio population. In addition, all dogs from San Antonio were younger, intact, and tended to be smaller which could have biased our metabolomic results. However, sterilization status did not have a significant effect on individual metabolites which is why it was removed from our final linear model analysis. Lastly, there was a difference in time spent fasting for the San Antonio compared with the other two locations as described in the methods. San Antonio dogs were fasted overnight while the same was not true for Birmingham and Columbia dogs. This difference in fasting could have contributed to some of the variations seen in the metabolome across the locations. Recent research in Labrador Retrievers suggests that the fasting plasma metabolome is significantly different from those of recently fed dogs (Soder et al. 2019). However, this study did not look at different periods of fasting. In addition, studies in humans suggest that only certain groups of metabolites (i.e., carnitines) are associated with fasting time (Sedlmeier et al. 2018). Overall, we conclude fasting status could have played a significant but likely minor role in our strong location signals. All these reasons notwithstanding, there are many other possibilities for the observed differences between locations including, but not limited to, different environmental exposures (e.g., diet, air quality, urban/rural residence, climate differences). Most likely, a combination of factors contributes to the strong location effect seen in our metabolomic samples.

Our study suggests that when working with individuals from different locations, especially if these are individuals living in the natural environment not the laboratory, we must control for potential location effects. The advice is most likely applicable to all “omics” in which “levels” of different biological factors are measured (i.e., transcriptomics and proteomics). Accordingly, any future canine studies from which samples are derived from different populations must have location as a controlling effect even if all sample handling procedures are consistent between sites.

Even with all the limitations of using non-laboratory animals in metabolomic profiling, we still find significant metabolites associated with age and weight in both our location residual analysis and our Birmingham-specific analysis. Metabolites associated with tryptophan metabolism and fatty acid metabolism were found to be differentially regulated between large and small dogs. This held true when looking at the entire dataset controlling for location effect and in the Birmingham-only dataset (the only dataset for which a large enough number of metabolites passed our FDR to be used for enrichment analyses). Metabolites in the tryptophan metabolism pathway tended to be higher in small dogs compared with large dogs (Figs. 3 and 4). We found no effect of weight on the two major players in the tryptophan metabolism pathway: l-tryptophan and kynurenine. However, no metabolites related to tryptophan metabolism showed a positive associated between weight and metabolite concentration. This pattern supports recent research suggesting that tryptophan metabolism may be an integral part of aging and longevity. High tryptophan (van der Goot and Nollen 2013) and low kynurenine (Sutphin et al. 2017) have been shown to promote longevity in worms, and low tryptophan levels were associated with increased risk of mortality in marmosets (Hoffman et al. 2018b).

The tryptophan metabolic network is complex. Tryptophan is a necessary precursor for the synthesis of the neurotransmitters, serotonin, and melatonin. However, 95% of dietary tryptophan enters the kynurenine pathway, where an end-product is NAD, a key player in energy metabolism and a co-factor for many enzymes such as sirtuins that are known to be involved in multiple aspects of health and longevity (Mouchiroud et al. 2013). Enhancing physiological NAD via various precursors has been shown to increase longevity and health in multiple species (Zhang et al. 2016). On the other hand, inhibition of the kynurenine pathway—which should reduce NAD—has also been observed to increase health and longevity (van der Goot and Nollen 2013). The resolution of this seeming paradox likely involves bioactive intermediates in the kynurenine pathway such as quinolinic acid which is an NMDA receptor *agonist* and/or kynurinic acid which is an *antagonist* of glutamate receptors. The kynurenine pathway also interacts with the mTOR network (Badawy 2017). Dogs, with their range of longevities, may be informative in refining our knowledge of the intricacies of this pathway.

Similar to our results, tryptophan itself was not found to be associated with weight in a previous canine metabolomics study (Middleton et al. 2017); however, they failed to find a signal of tryptophan degradation in their body weight analysis. Changes in tryptophan metabolism have also been shown to be related to diarrhea in dogs (Guard et al. 2015), suggesting changes in tryptophan metabolism might be indicative not just of size but also disease in individual dogs. While these studies combined with ours would suggest that higher tryptophan pathway metabolite levels are beneficial for longevity, previous research has shown that tryptophan restriction increases lifespan in both mice (De Marte and Enesco 1986) and rats (Segall and Timiras 1976). Therefore, the overall contribution of the tryptophan pathway to aging and longevity is still a very much needed area of research. Further interrogation of the tryptophan metabolism pathway is warranted to understand how manipulation may influence size and longevity.

Surprisingly, we failed to find any metabolite associated with weight or age in our Columbia population, even though they were more similar to the Birmingham population when comparing the entire metabolome. This population of dogs came from the Veterinary Health Center at the University of Missouri College of Veterinary Medicine, and as such represented an older, sicker population compared with the other two locations with the majority of dogs sampled in this population having at least one major health concern. These differences in health status are potentially driving the lack of metabolomic associations in the population and would also lend support to our PCA analysis which discovered some separation of a dogs diagnosed with and without cancer (Fig. 5). As older adults often present with at least one major morbidity, the Columbia results suggest that future large metabolomic studies in humans might also fail to find strong age or sex effects due to the overwhelming physiological changes that occur in response to disease as individuals age.

Interestingly, sex was not associated with individual metabolites. None passed our FDR correction threshold across locations, and only San Antonio showed a sex effect of any metabolites in the location-specific models. The San Antonio samples may have been characterized by differences by sex because they were all from intact dogs that were primarily young to middle-aged. Thus, the variation from sterilization and age was much less in this population compared with the other two locations. The overall lack of metabolites associated with sex is consistent with our previous finding that companion dogs do not show sex differences in longevity nor causes of death (Hoffman et al. 2017). However, these results are in contrast to the majority of the metabolomic literature which suggests that sex plays a large role on individual metabolites. Potentially, our FDR threshold was too stringent for the small effects of sex, but as we are looking at thousands of metabolites, these differences manifest themselves when combined together.

Our results combined increase knowledge to the overall field of metabolism and aging. Metabolism itself has been strongly linked to aging for more than a century (Austad and Fischer 1991; Rubner 1908). Over the past decade however, attention has focused more on the details of metabolic regulation which has emerged as a major contributor to the aging phenotype. Metabolomic studies have been increasingly used in both model (e.g., Fuchs et al. 2010; Hoffman et al. 2014; Houtkooper et al. 2011) and non-model (Ball et al. 2018; Hoffman et al. 2018b; Hoffman et al. 2016; Lewis et al. 2018; Viltard et al. 2019) organisms, leading to the discovery of common conserved pathways associated with aging and longevity. Also, diet as shown by dietary restriction, the most robust method to increase lifespan and health in model organisms, has been found to profoundly modify metabolic regulation (Matyi et al. 2018). Combined, it is evident that research on metabolic regulation with attention to individual metabolites may provide novel insights into mechanisms underlying the aging phenotype.

While we have presented the largest canine metabolome study to date, our results are not without many limitations due to the nature of sampling companion owned dogs. First, a large proportion of the metabolome is influenced by the environment—specifically the diet—and we do not know what each individual dog ate or was exposed to. However, we would not expect there to be strong differences across the three populations with regard to diet, as it would not be expected that owners in one location would feed a significantly different diet than another location within the USA. We would assume, though our assumption could be incorrect, that the variation in diets is the same across all three populations. While diet most likely does not explain the large differences between populations, diet is most assuredly leading to noise variation in the data. In addition, we lack information on the timing, amount, or type of food consumption for each individual dog, although any or all of these could be contributing to the changes we see in tryptophan metabolism between large and small dogs. Second, circadian rhythms play a large role in metabolomic profiles, and as these were personal owned dogs, we cannot control the time of day that each sample was taken. It was dependent on when the dog was brought into the clinic. Similar to the diet results, time of day of sample collection should not differ between the locations but would add more noise into our collected data. Third, while we do not know why our locations showed significantly different metabolomic profiles, some hypotheses are described above. However, there could have been differences in sample handling between the three locations that we are unaware of that led to the variation seen in the dataset. Lastly, we did not investigate breed-specific differences in our analysis, as we did not have the statistical power to do so with the number of breeds included in the study. Previous research, however, has shown that breeds do have different metabolomic profiles (Liu et al. 2016; Nicholatos et al. 2019), so breed variation in this study is likely to have influenced our metabolomic profiles. However, the wide range of breeds, including mixed-breed dogs, in our analysis actually allows us to better focus on size and age differences, as our results were significant despite the number of breeds represented in our study. Thus, the results reported here are likely not due to breed-specific differences but are more likely due to changes in age and genetic size effects.

## Conclusions

Here, we found a strong effect of location on metabolomic profiles in companion dogs; our results suggest that metabolomic profiles can be strongly influenced by location, and future large “omics” studies need to account for this strong geographic signal. After controlling for location effects, we found a strong signal of tryptophan metabolism and size. Tryptophan pathway metabolites were higher in small, long-lived dogs compared with their large counterparts, and future studies are needed to determine the direct physiological consequences of tryptophan metabolism manipulation. We still have ways to go to fully understand the metabolic differences that are found between large and small dogs, but we have paved the way for future large-scale “omics” studies in the companion dog.

## Electronic supplementary material

Figure S1. PCA of weight (A.) and age (B.) effects using location residuals in the positive ion mode. (PNG 340 kb)

High resolution image (TIFF 15689 kb)

Figure S2. Association of body weight with true tryptophan concentrations. (PNG 796 kb)

High resolution image (TIFF 30744 kb)

ESM 1 (XLSX 16 kb)
