# Supplementary figures and images for: Tryptophan metabolism is differently regulated between large and small dogs
Source: GeroScience. 2019 Nov 29;42(3):881–96. doi: 10.1007/s11357-019-00114-x (PMC7286990; doi:10.1007/s11357-019-00114-x)

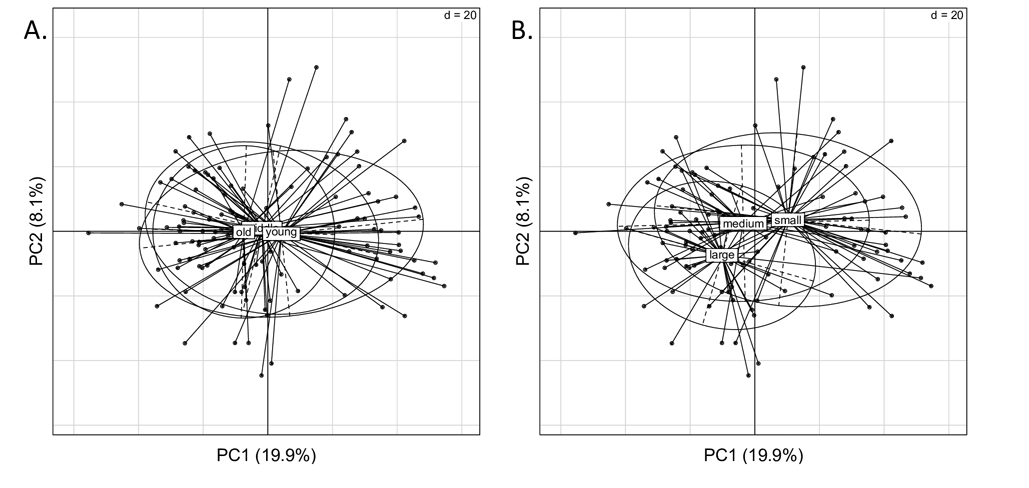

Supplement: Supplementary file 1 — PCA of weight (A.) and age (B.) effects using location residuals in the positive ion mode. (PNG 340 kb) [file 11357_2019_114_Fig6_ESM.png]

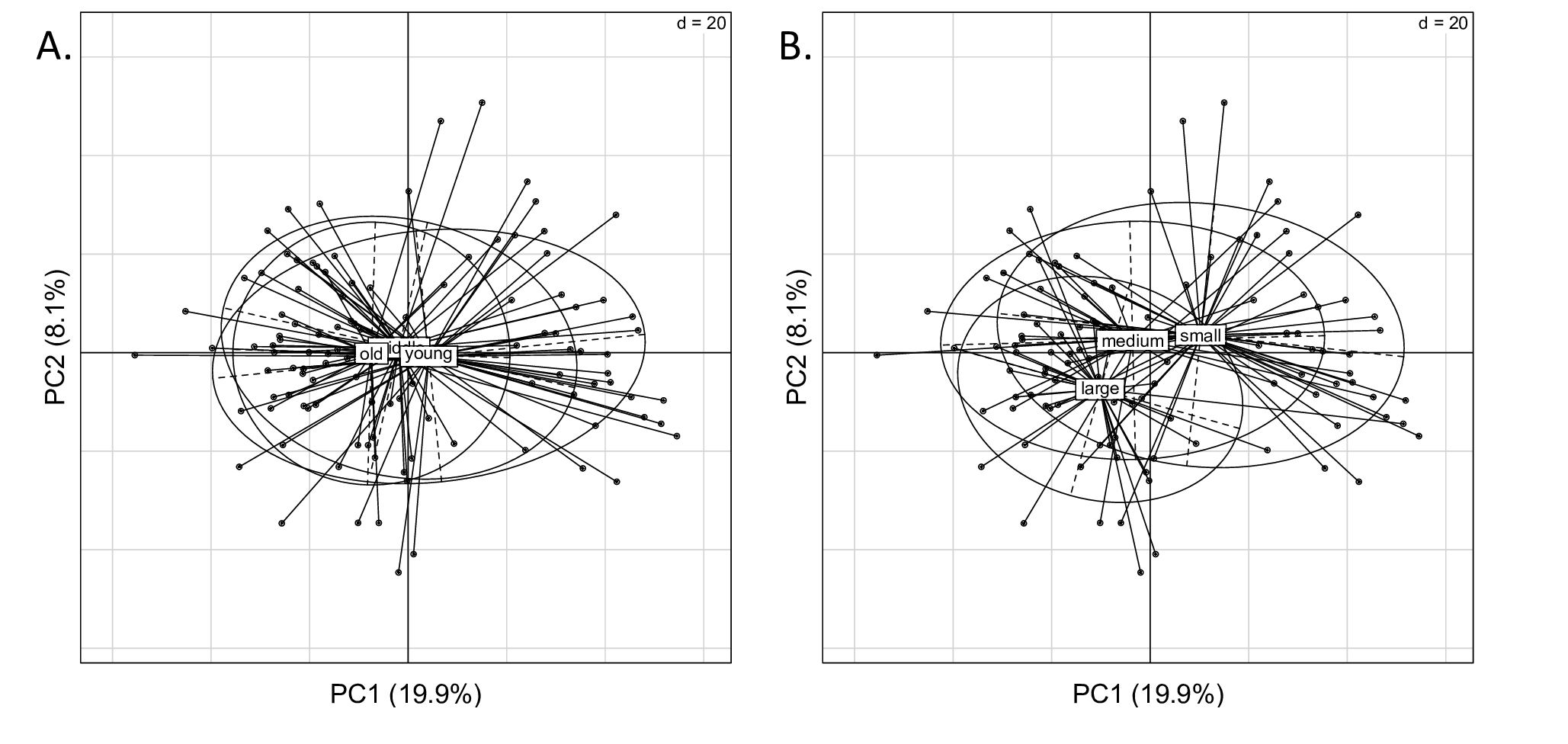

Supplement: Supplementary file 2 — High resolution image (TIFF 15689 kb) [file 11357_2019_114_MOESM1_ESM.tiff]

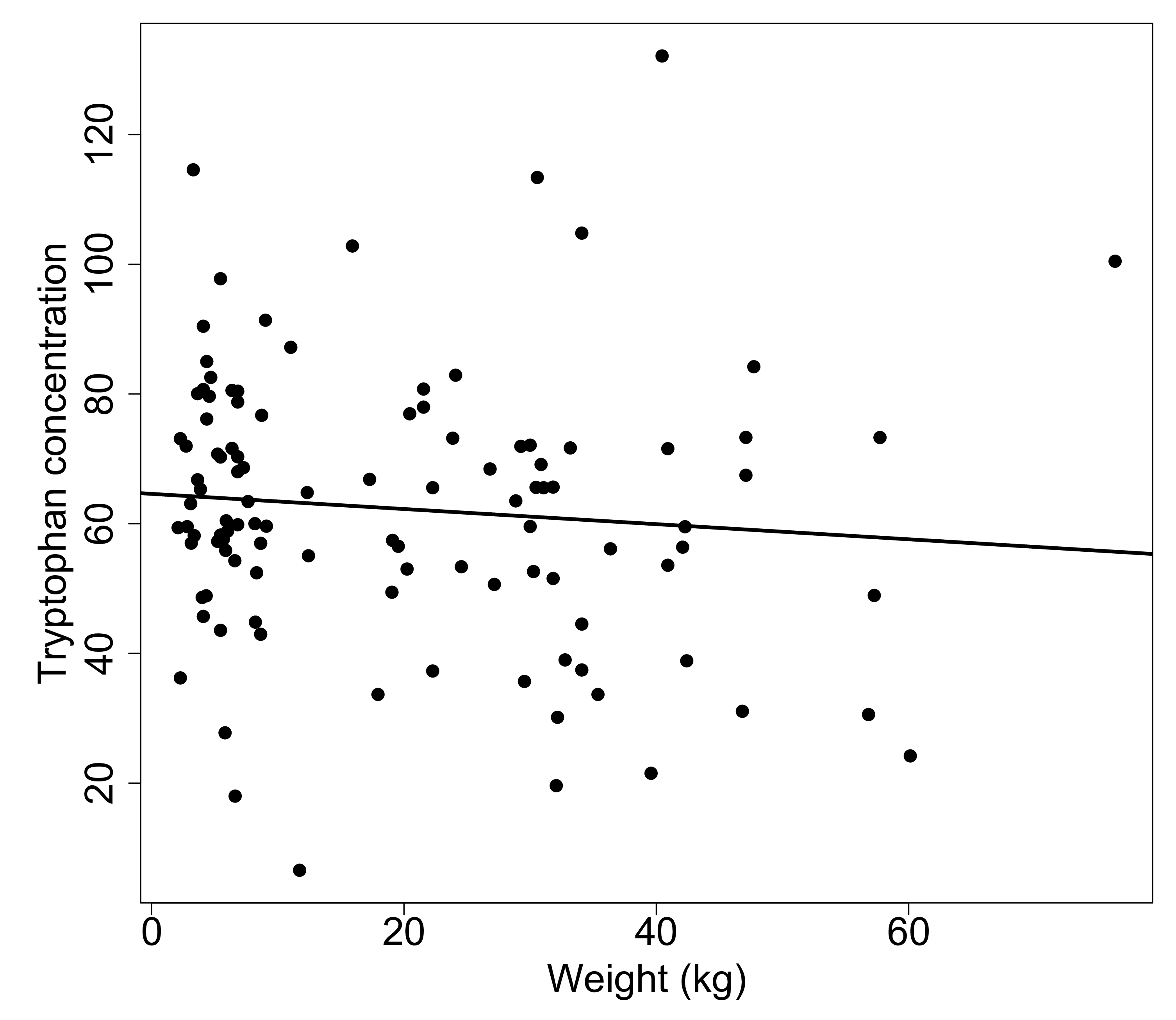

Supplement: Supplementary file 3 — Association of body weight with true tryptophan concentrations. (PNG 796 kb) [file 11357_2019_114_Fig7_ESM.png]
